# Fluctuations in Parkinson’s disease and personalized medicine: bridging the gap with the neuropsychiatric fluctuation scale

**DOI:** 10.3389/fneur.2023.1242484

**Published:** 2023-08-17

**Authors:** Emmanuelle Schmitt, Bettina Debu, Anna Castrioto, Andrea Kistner, Valerie Fraix, Martine Bouvard, Elena Moro

**Affiliations:** ^1^Division of Neurology, CHU Grenoble Alpes, Grenoble Institute of Neuroscience, INSERM U1216, Grenoble Alpes University, Grenoble, France; ^2^Psychology and Neurocognition Laboratory, Grenoble Alpes University, Université Savoie Mont Blanc, CNRS, LPNC, Grenoble, France

**Keywords:** psychometric characteristics, scale, validation, Parkinson‘s disease, neuropsychiatric fluctuations

## Abstract

**Background:**

Neuropsychiatric fluctuations (NpsyF) are frequent and disabling in people with Parkinson’s disease (PD). In OFF-medication, NpsyF entail *minus* neuropsychiatric symptoms (NPS) like anxiety, apathy, sadness, and fatigue. In ON-medication, NpsyF consist in *plus* NPS, such as high mood, hypomania, and hyperactivity. Accurate identification of these NpsyF is essential to optimize the overall PD management. Due to lack of punctual scales, the neuropsychiatric fluctuation scale (NFS) has been recently designed to assess NpsyF in real time. The NFS comprises 20 items with two subscores for *plus* and *minus* NPS, and a total score.

**Objective:**

To evaluate the psychometric properties of the NFS in PD.

**Methods:**

PD patients with motor fluctuations and healthy controls (HC) were assessed. In PD patients, the NFS was administrated in both the ON-and OFF-medication conditions, together with the movement disorders society-unified Parkinson disease rating scale parts I–IV. Depression (Beck depression scale II), apathy (Starkstein apathy scale) and non-motor fluctuations items of the Ardouin scale of behaviour in PD (ASBPD OFF and ON items) were also assessed. NFS internal structure was evaluated with principal component analysis consistency (PCA) in both medication conditions in PD patients and before emotional induction in HC. NFS internal consistency was assessed using Cronbach’s alpha coefficient. NFS convergent and divergent validity was measured through correlations with BDI-II, Starktein, and ASBPD OFF and ON non motor items. Specificity was assessed comparing NFS global score between the HC and PD populations. Sensitivity was evaluated with t-student test comparing the ON-and the OFF-medication conditions for NFS global score and for *minus* and *plus* subscores.

**Results:**

In total, 101 consecutive PD patients and 181 HC were included. In PD patients and HC, PCA highlighted one component that explained 32–35 and 42% of the variance, respectively. Internal consistency was good for both the NFS*-plus* (alpha =0.88) and NFS*-minus* items (alpha =0.8). The NFS showed a good specifity for PD (*p <* 0.0001) and a good sensitivity to the medication condition (*p <* 0.0001).

**Conclusion:**

The satisfactory properties of the NFS support its use to assess acute neuropsychiatric fluctuations in PD patients, adding to available tools.

## 1. Introduction

Non-motor fluctuations are frequent in advanced Parkinson’s disease (PD) and have a major negative impact on patients’ autonomy and quality of life (1, 2). Indeed, disability linked to non-motor fluctuations can be greater than that caused by motor fluctuations (3).

Neuropsychiatric fluctuations (NpsyF) are possibly the most disabling non-motor fluctuations (4–7). In the OFF-medication condition, NpsyF are characterized by *minus* neuropsychiatric symptoms (NPS), also called hypo-dopaminergic symptoms, like anxiety, fatigue, lack of motivation, sadness, and slowness of thinking. Conversely, in the ON-medication condition, *plus* NPS, also called hyper-dopaminergic symptoms, can be described by a general feeling of well-being, high mood (even hypomania), and hyperactivity (2). The presence and severity of NpsyF can differ from one patient to another, likely depending on the extent of central dopaminergic depletion and sensitivity of D_3_-D_5_ receptors of the mesocorticolimbic pathway (8). NpsyF are also considered to be a risk factor for developing dopaminergic dysregulation syndrome or hypo-dopaminergic syndrome after deep brain stimulation (DBS) of the subtalamic nucleus (STN) (6, 9). Prompt and careful identification and management of these NpsyF play an essential role in the overall management of PD.

Although several retrospective questionnaires and scales are currently available to measure non motor fluctuations in people with PD (6, 10–15), none is specific to punctually assess the presence and severity of NpsyF. To fill this fundamental gap, our group has developed the neuropsychiatric fluctuation scale (NFS) (16). In 18 PD patients with motor fluctuations, we found a positive correlation between *NFS-minus* subscore and motor symptoms in the OFF medication condition, namely bradykinesia (17). The *NFS-minus* and *NFS-plus* subscores also inversely correlated with each other, depending on the medication condition. These findings suggest that the NFS can capture opposite conditions and identify both the ON and OFF non motor symptomes. Although tested in a small sample, the NFS was easily applied, and results matched the expected findings.

To date, the validation of the NFS is ongoing in France (ClinicalTrials.gov Identifier: NCT04455074), and it will be soon completed.

The aim of this study was to further analyze the psychometrics qualities of the NFS scale. We focused on the internal structure of the scale to identify the number of dimensions constituting the NFS, and the way the items are distributed across these dimensions. We studied the internal consistency, i.e., the degree of homogeneity among the items of the NFS, assessing whether they were consistent with one another and measured the same aspect. We also looked at the convergent and divergent valididity, i.e., the degree to which a measure is related to or divergent from another measure of which the underlying construct is conceptually related or unrelated. The specificity, i.e., the ability of the NFS to differentiate the PD population from the HC, and the sensitivity, i.e., the ability of the NFS to detect NpsyF, were also analyzed.

## 2. Methods

### 2.1. Participants and data collection

We collected retrospective data from PD patients hospitalised for evaluations before STN DBS surgery at the Movement Disorders center in the Centre Hospitalier Universitaire Grenoble Alpes (CHUGA) of Grenoble, France, from September 2016 to June 2021. Inclusion criteria were: diagnosis of idiopathic PD (18), presence of motor fluctuations, and no dementia.

Healthy controls (HC) were recruited from the general population through advertisements in companies, social networks, and caregiver associations. Inclusion criteria were: age between 40 and 75 years, and the absence of neurological or psychiatric disorders. All HC assessments were conducted online.

### 2.2. Ethical considerations

The Department of Clinical Research and Innovation (DRCI) of the CHUGA gave its approval for this research. PD patients have been fully informed of the objectives of the study and the nature of the informations collected, including their right to object at any time to the use of collected data. For HC, the ethics committee for research and teaching of the University of Savoie Mont Blanc approuved the study, and participants completed a consent form before starting the study.

### 2.3. Procedure and measures

All PD patients had an acute levodopa challenge after an overnight medication withdrawal (19). The NFS (see below) was administrated in both the OFF-and ON-medication conditions along with motor examination, using the movement disorders society-unified Parkinson disease rating scale (MDS-UPDRS) part III. Non motor signs (MDS-UPDRS part I), activities of daily living (MDS-UPDRS part 2), and motor complications (MDS-UPDRS part IV) were also collected (20). In the ON condition, patients also filled the Beck depression inventory (BDI-II) (21), the Starkstein apathy scale (22), and the non-motor fluctuations items of the Ardouin Scale for Behavioral Assessment in Parkinson’s Disease (ASBPD OFF and ON items) (23, 24). The cognitive global state and the executive functions were evaluated using the Mattis dementia rating scale (MDRS) (25) and the frontal score (26).

The HC group filled the NFS before and after a simple emotional induction. Since spontaneous and brief emotional fluctuations do not exist in the general population, they were artificially induced through an emotional induction task. The emotional induction procedure consisted of viewing 3 min video clips from the StimFilm database (27). Participants were randomly assigned to one of three experimental conditions (positive, negative, or neutral emotion induction). In addition, mood condition was examined with the Beck depression inventory (BDI-II).

Demographic data (age and gender for patients and HC), PD duration, and levodopa equivalent daily dosage (LEDD) were also recorded.

**Figure 1:**
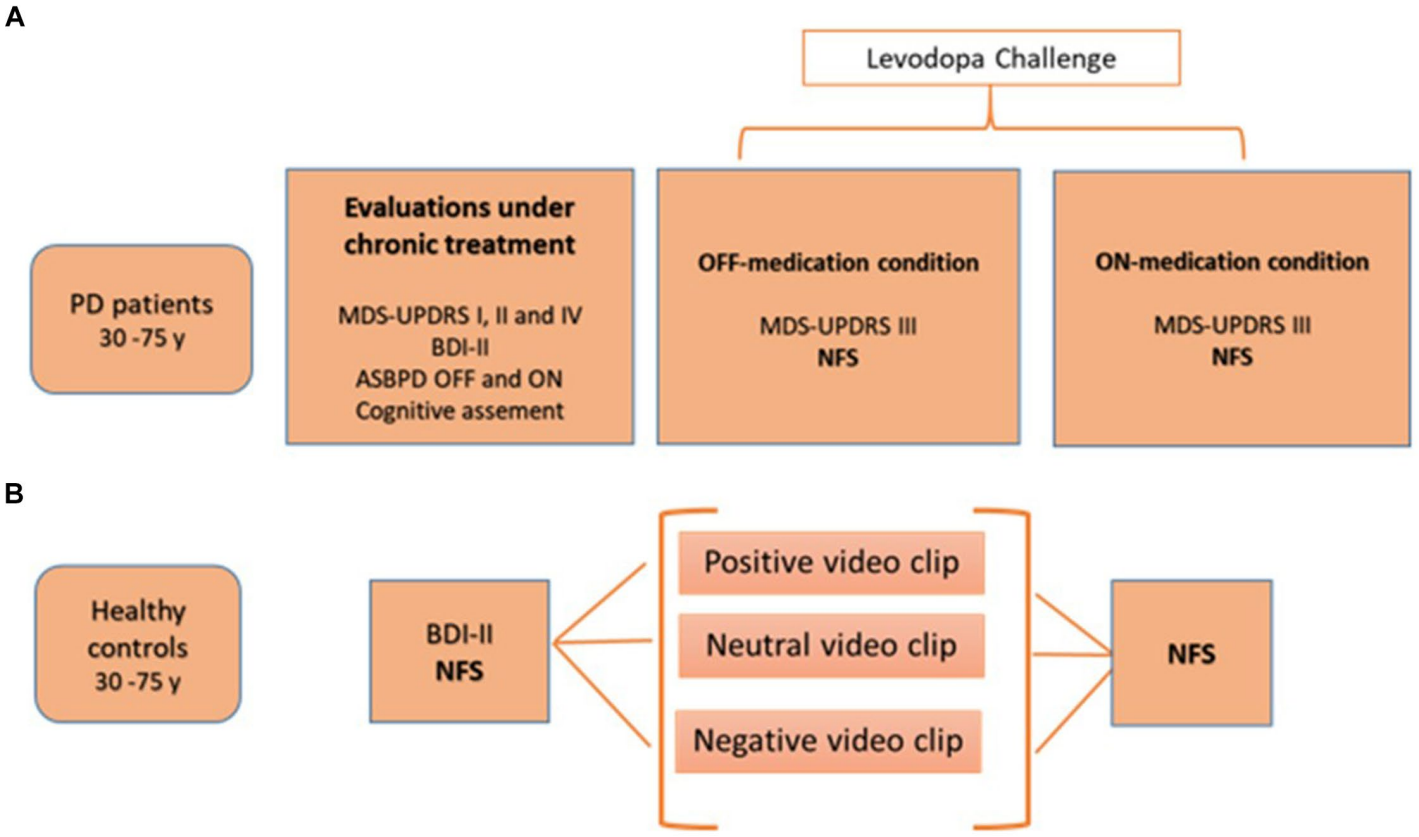
Study design for PD patients **(A)** and healthy controls **(B)**. ASBPD, Ardouin scale for behavioral assessment in Parkinson’s disease; BDI-II, Beck depression inventory; MDS-UPDRS, movement disorders society-unified Parkinson disease rating scale; NFS, neuropsychiatric fluctuation scale; PD: Parkinson’s disease.

Figure 1 shows the study design.

### 2.4. The NFS

**Figure 2:**
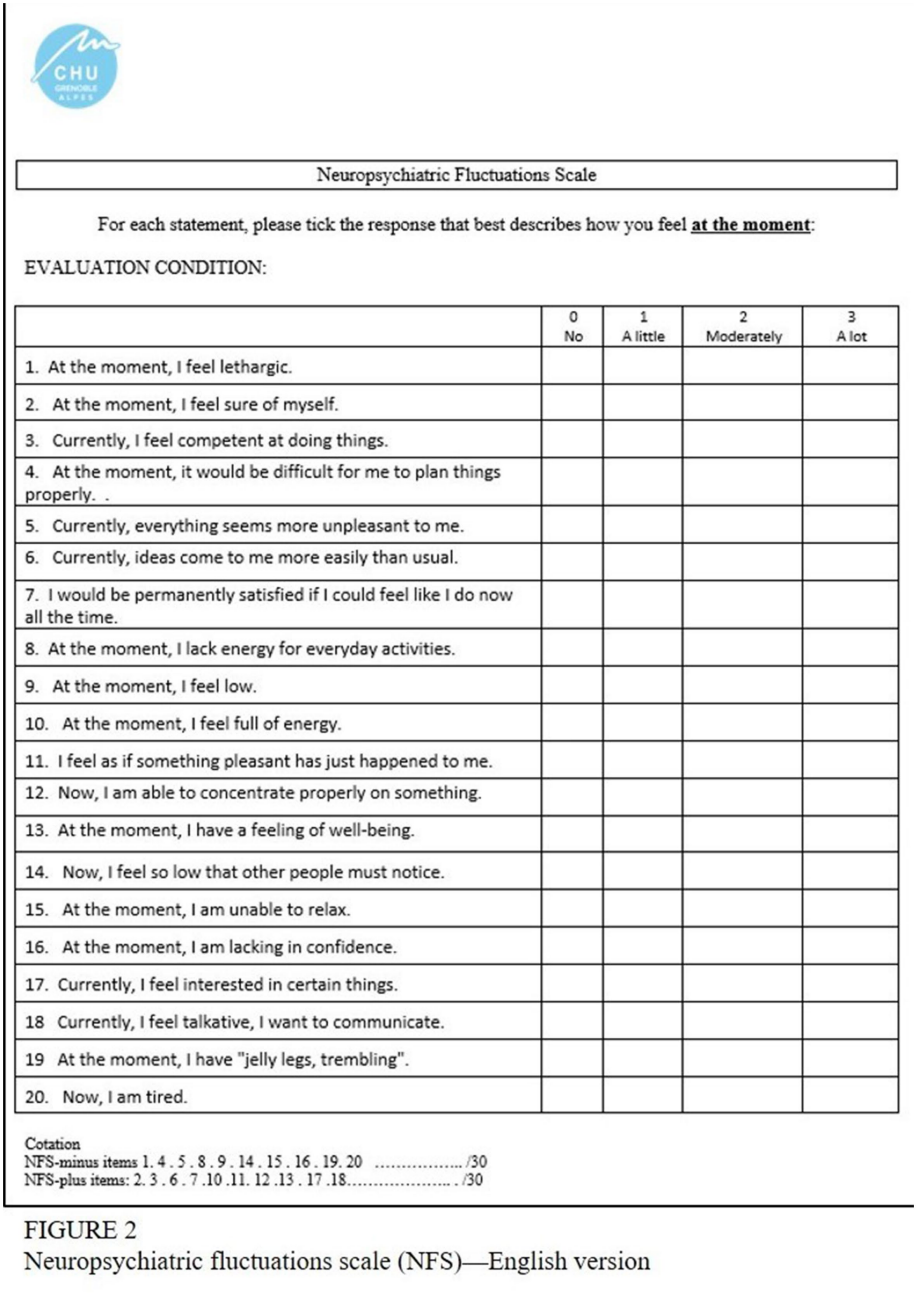
Neuropsychiatric fluctuations scale (NFS)—English version.

Briefly, the NFS is composed by 20 items, divided in two parts: the NFS*-plus* and the NFS*-minus* (16). The NFS*-plus* part includes NFS hyper-dopaminergic symptoms, i.e., items describing patients’ feelings in the ON-medication condition, such as euphoria and wellbeing. In the scale, the NFS*-plus* items are number 2, 3, 6, 7, 10, 11, 12,13, 17, and 18. The NFS*-minus* part corresponds to hypo-dopaminergic symptoms expressed during the OFF-medication condition, i.e., apathy, anxiety, attention issues, depression, and fatigue. In the scale, the NFS*-minus* items correspond to number 1, 4, 5, 8, 9, 14, 15, 16, 19, and 20. The NFS can be completed in the OFF-and the ON-medication condition. The scale provides two subscores with a maximal total score of 30 points each. The global score ranges from −30 to +30. A negative global score means that the OFF neuropsychological symptoms are predominant, whereas a positive global score reflects a majority of ON-neuropsychological symptoms. See Figure 2 for further details.

### 2.5. Statistical analysis

The main clinical and neuropsychological characteristics of PD patients and HC were described using means, standard deviations, and max-min data. The two groups were compared by age and depression criteria using *t*-tests. The normality of distribution for each variable was verified using Shapiro–Wilk test.

Analysis of the internal structure of the questionnaire was performed using a principal component analysis (PCA) with an Oblimin rotation. For PD patients, PCA was applied on global scores in each medication condition; for HC, PCA was applied on the global score before emotional induction.

The internal consistency of the scale was assessed by calculating the Cronbach’s *alpha* for global scores for HC and the patients (in both the ON-and OFF-medication conditions). Based on the usual recommendations, a 0.70–0.90 outcome was considered a good internal consistency (28).

Convergent and divergent validity of the NFS was measured using Spearman correlations for BDI-II, Starkstein apathy scale, and ASBPD non motor items. In the OFF-medication condition, we hypothesized that the *minus* subscore was positively correlated with the depression and apathy scales, and the ASBPD OFF item. Conversely, in the ON-medication condition, we expected that the *plus* subscore would be negatively correlated with these same psychologic scales.

The specificity of the NFS was evaluated by NFS global scores comparisons between: (a) HC after positive induction (HC^+^) and PD patients in the ON-medication condition; (b) HC after negative induction (HC^−^) and PD patient in the OFF-medication condition. The Mann Whitney test was used. We hypothesized that NFS global scores between PD patients and HC would be significantly different for each comparison.

Sensitivity of the NFS was assessed by comparing the ON-and the OFF-medication conditions for NFS global scores, and for *minus* and *plus* subscores using the Student test. We expected a significant difference between the NFS global scores in each medication condition, and also between the *plus* and *minus* subscores in each condition.

Data were analyzed using JASP software, version 0.16.4 (intel).

## 3. Results

### 3.1. Clinical characteristics

A total of 101 consecutive PD patients were included from September 2016 to June 2021. The main clinical characteristics of PD patients are detailed in Table 1.

**Table 1:** Main clinical and neuropsychological characteristics of people with Parkinson’s disease (PD).

	Subjects (n.)	Mean (SD); max–min
**Clinical assessment**		
PD duration (years)	101	9.6 (3.6); 21–4
LEDD total (mg)	101	1307.0 (467); 2,715–450
MDS-UPDRS III OFF-med	101	45.2 (16.08); 102–16
L-dopa challenge (mg)	101	282.9 (76.7); 500–100
MDS-UPDRS III ON-med	101	16.7 (8.9); 47–3
MDS-UPDRS I	98	15.6 (6.6); 34–1
MDS-UPDRS II	98	15.6 (5.8); 30–4
MDS-UPDRS IV	98	9.8 (3.35); 20–3
Item 4.3 (time in OFF)	100	1.56 (0.7); 4–0
**Neuropsychological assessment**		
ASBPD OFF	92	1.56 (0.7); 4–0
ASBPD ON	92	1.78 (1.16); 3–0
BDI-II	82	12.4 (7.5); 37–1
Stakstein	70	10.8 (4.9); 24–0
MDRS	86	138.2 (4.88); 144–117
Frontal score	97	41.4 (8.42); 50–15

ASBPD, Ardouin scale for behavioral assessment in Parkinson’s disease; BDI-II, Beck depression inventory; LEDD, levodopa equivalent daily dose; MDRS, Mattis dementia rating scale; PD, Parkinson’s disease; SD, standard deviation.

One hundred and eighty-one HC were included from January 2020 to November 2022.

Average age and mood condition were significantly different (*p <* 0.001) between the two groups. Table 2 shows PD and HC clinical caracteristics comparaison.

**Table 2:** Comparaison between PD and HC characteristics.

	PD patients		HC		*t*	*p*
	*N*	Mean (SD); max–min	*N*	Mean (SD); max–min		
Sex (M/F)	101	63/38	181	59/122		
Age (years)	101	61.0 (7.5); 79–41	181	56.0 (9.2); 78–40	−3.849	<0.001
BDI-II	82	12.4 (7.5); 37–1	181	7.1 (7.1); 35–0	−5.487	<0.001

BDI-II, Beck depression inventory; HC, healthy controls; PD, Parkinson’s disease; SD, standard deviation.

### 3.2. Psychometric characteristics

The NFS global scores did not follow the normal distribution, neither in the HC group (*W =* 0.94; *p <* 0.0001) nor in the PD group (OFF-medication condition: *W =* 0.94; *p =* 0.002/ON-medication condition: *W =* 0.95; *p =* 0.008).

#### 3.2.1. Internal structure

PCA performed in PD patients in the OFF-and the ON-medication conditions, and in HC before emotional induction highlighted one dimension that explained 32, 39 and 42% of the variance, respectively.

In the OFF-medication condition, items 1, 4, 5, 8, 9, 14, 15, 16, 19, and 20 (corresponding to NFS-*minus* items) were positively related to this unique dimension. On the contrary, items 2, 3, 6, 7, 10, 11, 12, 17, and 18 (corresponding to *NFS-plus* items) were negatively related to this dimension. Item 13 showed a poor saturation in this dimension (<30).

Conversely, in the ON-medication condition, items 2, 3, 6, 7, 10, 11, 12, 13, 17, and 18 were positively related to the dimension, whereas items 1, 4, 9, 14, 15, 16, 19, and 20 were negatively related. Items 5 and 8 showed a poor saturation in this dimension (<30).

In HC, before emotional induction, items 2, 3, 6, 7, 10, 11, 12, 13, 17 and 18 were positively related to the unique dimension, whereas items 1, 4, 5, 8, 9, 14, 15, 16, and 20 were negatively related. Item 19 showed a poor saturation in this dimension (<30). The items’ distribution in HC was the same as that observed in PD patients in the ON-medication condition.

Table 3 shows the saturation of the NFS items in PD patient in the OFF-and ON-medication conditions, and in HC.

**Table 3:** Saturation of the NFS items for PD patient in the OFF-medication condition (PD OFF-med), and the ON-medication condition (PD ON-med), and for HC.

NFS items	Items’saturation (RC)		
	PD OFF-med	PD ON-med	HC
1	0.499	−0.626	−0.604
**2**	**−0.682**	**0.714**	**0.680**
**3**	**−0.740**	**0.752**	**0.513**
4	0.355	−0.603	−0.516
5	0.639	−0.269	−0.474
**6**	**−0.387**	**0.461**	**0.402**
**7**	**−0.466**	**0.802**	**0.668**
8	0.634	−0.293	−0.668
9	0.724	−0.602	−0.574
**10**	**−0.531**	**0.690**	**0.881**
**11**	**−0.458**	**0.642**	**0.701**
**12**	**−0.505**	**0.632**	**0.626**
**13**	**−0.108**	**0.837**	**0.848**
14	0.640	−0.615	−0.338
15	0.434	−0.486	−0.618
16	0.699	−0.737	−0.533
**17**	**−0.471**	**0.426**	**0.601**
**18**	**−0.398**	**0.526**	**0.649**
19	0.582	−0.543	−0.236
20	0.630	−0.564	−0.603
Eigenvalue	6.046	5.426	7.42
Proportion variance (%)	34	39	42

NFS-plus items are in bold; items’saturation <30 is highlighted in grey. NFS, neuropsychiatric fluctuations scale; PD, Parkinson’s disease.

#### 3.2.2. Internal consistency

In the OFF-and ON-medication conditions, the Cronbach’s *alpha* coefficient for EFN NFS global scores was 0.88 and 0.89, respectively. For HC, the Cronbach’s *alpha* coefficient was 0.93.

#### 3.2.3. Convergent and divergent validity

In the OFF-medication condition, *plus* subscores did not show significant correlations with emotional scales (apathy, depression, and OFF non-motor fluctuations) whereas *minus* subscores showed a positive correlation with OFF ASBPD (*p* = 0.004) and with BDI (*p =* 0.04), but no correlation with apathy. In the ON-medication condition, only the Starkstein apathy scale correlated negatively with *plus* subscores (*p =* 0.0005).

In HC, *minus* subscores strongly correlated with the BDI-II (*p <* 0.001) whereas *plus* subscores were negatively correlated with the BDI-II (*p <* 0.001).

#### 3.2.4. Specifity

**Figure 3:**
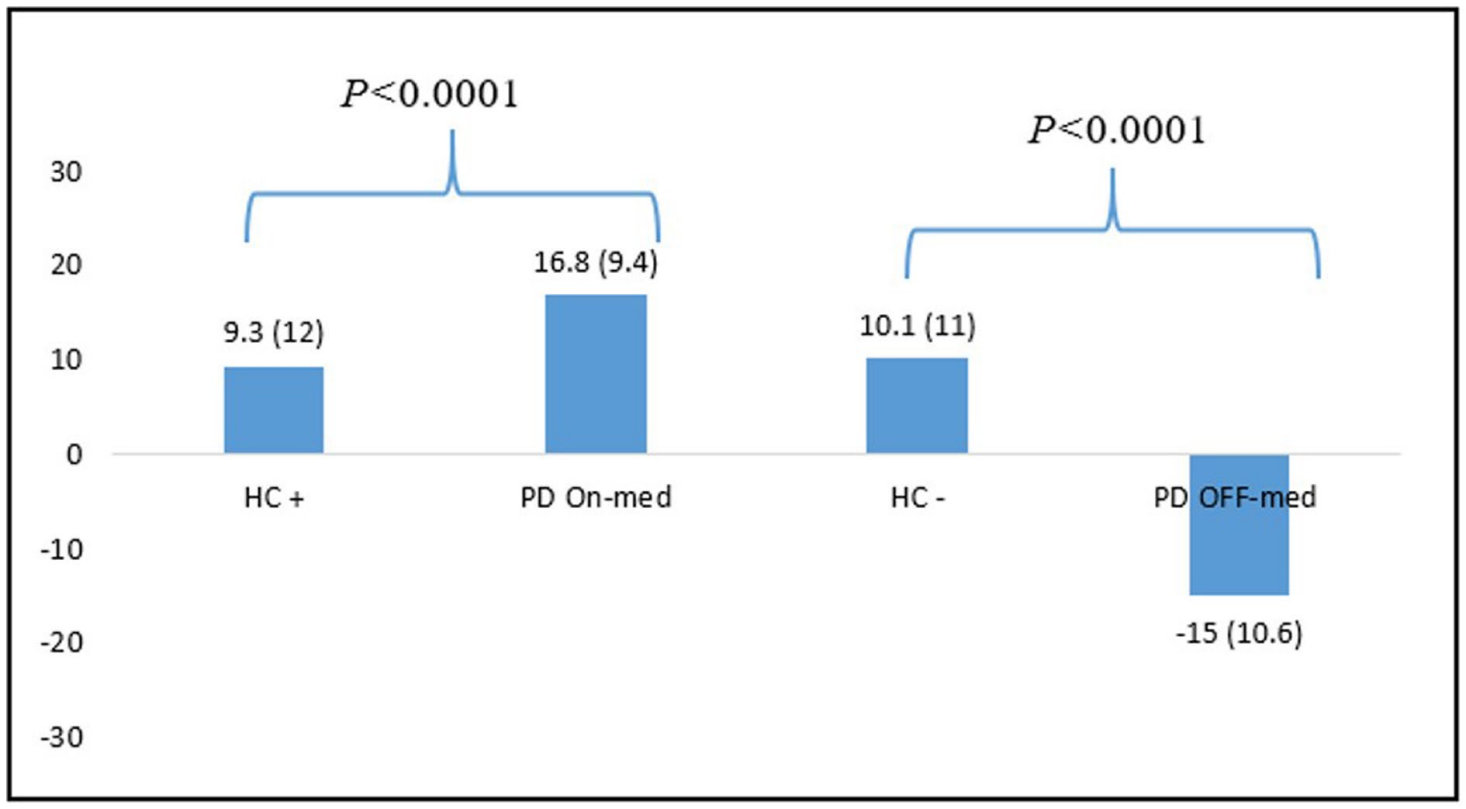
Differences between NFS global scores means for HC and PD patients in the ON-and OFF-medication conditions. HC, healthy controls; PD: Parkinson’s disease.

The NFS global scores comparison between HC^+^ (*N* = 54) and PD patients in the ON-medication condition, and between HC^−^ (*N* = 83) and PD patients in the OFF-medication condition showed significant differences between the two populations in both condition (*p <* 0.0001). See Figure 3 for more details.

#### 3.2.5. Sensitivity

NFS*-minus*, NFS*-plus*, and NFS global scores all significantly differed between the OFF-and the ON-medication conditions (all *p <* 0.001). For details, see Table 4.

**Table 4:** Student’ test values between the NFS subscores and global scores in the ON-and OFF-medication conditions in PD patients.

	Subjects (n.)	Mean (SD)	*t*	*p*
NFS global score OFF	101	−15 (10.6)	−21	<0.001
NFS global score ON	101	16.8 (9.4)		
NFS ON subscore plus	101	21 (6.3)	20.1	<0.001
NFS OFF subscore plus	101	4.6 (4.4)		
NFS ON subscore minus	101	4.2 (4.4)	−18.7	<0.001
NFS OFF subscore minus	101	19.5 (7.2)		

NFS, neuropsychiatric fluctuations scale; PD, Parkinson’s disease; SD, standard deviation.

## 4. Discussion

The NFS has been designed to acutely detect and quantify subjective changes in neuropsychological conditions in PD patients with motor fluctuations, especially in acute settings (16). In our study, we have demonstrated that the NFS is sufficiently sensitive to measure neuropsychiatric variations related to dopaminergic status, specifically in PD patients.

This pre-validation of the psychometric qualities of the NFS shows that the internal structure of our tool, measured with the PCA, revolves around a single dimension, with some items positively and others negatively related to the dimension. For PD patients, the distribution of items that change according to the ON and OFF-medication conditions underlines that this unique dimension is probably the dopaminergic state. Therefore, the items’distribution in the OFF-medication condition reflects the hypo-dopaminergic state, whereas the items’ distribution in the ON-medication condition reflects the hyper-dopaminergic state. Several studies showing the effects of L-dopa treatment on mood, motivation, and also cognitive changes in non-demented patients have supported a dopaminergic involvement in NpsyF (29–32). NpsyF are likely linked to changes in the dopaminergic mesolimbic pathway. When the mesolimbic dopamine concentration is low, OFF-NpsyF (fatigue, depression, low motivation, and slowness of thinking) occur (33, 34). In contrast, the increase in dopamine concentration after dopaminergic drugs intake is associated with ON-NpsyF (feeling of well-being, increased motivation, better attentional functions), and also hyper-dopaminergic behaviors (35).

The PCA performed on HC shows the same items’ distribution as observed in PD patients in the ON-medication condition. The items of the NFS are also distributed around a single dimension. This shows consistency in the construction of the scale.

In PD population, the NFS global scores demonstrated a good internal consistency in both the OFF-and ON-medication conditions (*alpha >*0.80). The NFS internal consistency was also very good in HC (*alpha >*0.90). These results indicate that the items of the scale are sufficiently interconnected and measure the same construct.

For convergent and divergent validity, the correlations between NFS*-plus* and-minus subscores, scales measuring depression (BDI-II), apathy (Starktein), and a tool measuring NFpsy (OFF and ON ASBPD) show that, in the OFF-medication condition, the *minus* subscore is linked to the mood assessment and another scale measuring OFF dysphoria (ASBPD OFF), whereas the *plus* subscore is negatively correlated with depression. In the ON-medication condition, we found a negative correlation between the *plus* subscore and the scale measuring apathy. This suggests that the *minus* subscore highlights OFF dysphoria when PD patients are dopamine deprived, whereas the *plus* subscore reflects increased motivation and activity seeking when PD patients are under dopaminergic stimulation. This is consistent with previous studies describing NFpsy characteristics (7, 8, 36). We also found this pattern of correlation in HC in whom *minus* subscore correlated with the depression scale, and a *plus* subscore negatively correlated on the same measure. This confirms that the NFS’s subscores can reflect two opposite mood states even in HC.

We also demonstrated that the NFS global score allows to properly differentiate HC from the PD patients in each condition. PD patients showed significantly higher scores than the control population. In the OFF-medication condition, patients showed very negative scores, reflecting a hypo-dopaminergic mood, whereas HC still had positive overall scores, showing a stable mood despite the induction. Conversely, in the ON-medication condition, patients had significantly more positive scores than HC after positive induction. These findings show that the NFS captures hyper-dopaminergic euphoria or hypomania specific to fluctuating PD patients, whereas HC stand in a standard positive mood even after positive induction. These results are consistent with data from the literature showing that the prevalence of mood disorders (depression, dysphoria, anxiety) and hypomania is greater in PD than in the general population (37, 38).

Our study has several limitations. First, the HC population and PD participants differed in terms of age and mood condition, which may limit the comparison between the two groups. Moreover, some concerns about the effectiveness of the emotional induction procedure chosen for the HC may arise. Although the presentation of emotional videos guarantees a more robust induction over time than presentation of emotional pictures, we cannot ensure that a single viewing was sufficient to induce a relevant emotional change (39). Furthermore, for the negative-valence videos, a medium valence was chosen in agreement with the ethical committee, to guarantee psychic safety to the participants, which may have contributed to diminishing the effect of the induction. Additionally, although our scale aims to assess acute mood changes, it lacks the evaluation of test-retest stability to have a complete study of the psychometric qualities of the scale.

Nevertheless, our pre-validation study shows that the NFS is an acceptable tool for detecting and evaluating NpsyF in PD. The NFS allows to define different profiles of fluctuating patients: patients with predominant OFF neuropsychiatric symptoms (who experience major dysphoria when treatment is ineffective, and return to a state of emotional homeostasis, without hypomania, when treatment is effective), patients with predominant ON neuropsychiatric symptoms (euphoric mood, compulsive and hyperactive behaviours in the ON phase, and a stable, non-depressive mood in the OFF phase), and also patients with both ON and OFF severe neuropsychiatric symptoms (moving from dysthymic to hypomanic periods, with few intervals of mood stability).This distinction can allow to optimize the medical treatment and overall management of different phenotypes of PD.

The NFS can also be used in chronic situations to detect neuropsychiatric profiles such as hypo-dopaminergic or hyper-dopaminergic syndromes. Overall, we can use the NFS in the same way and in addition to MDS-UPDRS part III, either to capture a chronic condition or to highlight fluctuations by repeating the measurements under different conditions.

## 5. Conclusion

Neuropsychiatric fluctuations are frequent in PD. These changes in mood and cognitive status experienced by PD patients severely influence their quality of life and experience of the disease. The NFS seems to be a reliable and handy tool for measuring NpsyF in acute setting in the PD population, thus helping physician to better personalize the patients’ management.

## Data availability statement

The raw data supporting the conclusions of this article will be made available by the authors, without undue reservation.

## Ethics statement

The studies involving humans were approved by The Department of Clinical Research and Innovation (DRCI) of the CHUGA and ethics committee for research and teaching of the University of Savoie Mont Blanc. The studies were conducted in accordance with the local legislation and institutional requirements. The participants provided their written informed consent to participate in this study.

## Author contributions

ES, VF, AK, and AC: data collection. ES, BD, MB, and EM: conception, design of the work, data analysis, interpretation, and drafting the article. ES, MB, and EM: critical revision of the article and final approval of the version to be published. All authors contributed to the article and approved the submitted version.

## Acknowledgments

The authors thank to all PD patients for giving their consent the use their medical data. The authors also thank the HC for their participation to our study. The authors thank Paul Krack and Pablo Martinez-Martin for their contribution to the development of the preliminary version of the Neuropsychiatric Fluctuation Scale (not used in this study).

## Conflict of interest

EM has received honoraria from Medtronic for consulting services. She has also received research grant support from the Grenoble Alpes University, Abbott, Ipsen, and France Parkinson.

The remaining authors declare that the research was conducted in the absence of any commercial or financial relationships that could be construed as a potential conflict of interest.

## Publisher’s note

All claims expressed in this article are solely those of the authors and do not necessarily represent those of their affiliated organizations, or those of the publisher, the editors and the reviewers. Any product that may be evaluated in this article, or claim that may be made by its manufacturer, is not guaranteed or endorsed by the publisher.
